# Burden of Disease and Treatment Patterns in Adults with Atopic Dermatitis from the Baltic Region: Real-World Data from the ESSENTIAL AD Cross-Sectional Study

**DOI:** 10.3390/medicina62010084

**Published:** 2025-12-31

**Authors:** Maigi Eisen, Brigita Gradauskiene, Jurate Grigaitiene, Ilona Hartmane, Külli Kingo, Ingmars Mikazans, Liisi Raam, Karin Toomela

**Affiliations:** 1 Center of Dermatology and Venereology, The North Estonia Medical Centre, 13419 Tallinn, Estonia; maigi.eisen@regionaalhaigla.ee; 2 Department of Immunology and Allergology, Lithuanian University of Health Sciences, 50161 Kaunas, Lithuania; brigita.gradauskiene@lsmu.lt; 3 Institute of Clinical Medicine, Clinic of Infectious Diseases and Dermatovenereology, Faculty of Medicine, Vilnius University, 03101 Vilnius, Lithuania; jurate.grigaitiene@mf.vu.lt; 4 Department of Dermatology and Venereology, Faculty of Medicine, Riga Stradins University, LV-1010 Riga, Latvia; ilona.hartmane@rsu.lv (I.H.); ingmars.mikazans@rsu.lv (I.M.); 5 Department of Dermatology and Venereology, University of Tartu, 50090 Tartu, Estonia; kylli.kingo@kliinikum.ee (K.K.); liisi.raam@kliinikum.ee (L.R.); 6 Dermatology Clinic, Tartu University Hospital, 50417 Tartu, Estonia; 7 AbbVie OÜ, 10145 Tallinn, Estonia

**Keywords:** atopic dermatitis, disease burden, quality of life, treatment patterns, real-world study, Baltic region

## Abstract

*Background and Objectives*: Nationwide registries that provide comprehensive insights into the atopic dermatitis (AD) population and management in routine practice are lacking in Baltic countries. Real-world studies to explore the clinical and economic burden of AD are highly needed. We present findings from the Baltic cohort of the larger observational study ESSENTIAL AD, conducted in Europe, the Middle East, and Africa. *Materials and Methods*: This cross-sectional, retrospective chart review study enrolled adult AD patients routinely managed with systemic and/or non-systemic therapy in Estonia, Latvia, and Lithuania. Data was collected during one office visit. AD severity was assessed using the Eczema Area and Severity Index (EASI) and SCORing Atopic Dermatitis (SCORAD) and impact on quality of life was assessed using the Dermatology Life Quality Index (DLQI) (primary endpoints). *Results*: Fifty patients were enrolled, with a mean (standard deviation [SD]) age of 33.6 (11.67) years, and 60% were women. Mean (SD) time since AD diagnosis was 21.8 (14.8) years. An equal proportion of patients received systemic therapy (including combination therapy) or non-systemic therapy (50% each). Mean (SD) EASI, SCORAD, and DLQI total scores were 9.8 (9.76), 38.0 (16.5), and 10.5 (7.1), respectively. No significant difference was observed between patients receiving systemic and non-systemic therapy in terms of EASI (mean [SD] 11.5 [12.2] versus 8.2 [6.3]; *p* = 0.7636), SCORAD (35.4 [20.8] versus 40.6 [11.5]; *p* = 0.2563), and DLQI (9.5 [7.6] versus 11.5 [6.5]; *p* = 0.1962). Hospitalization rate (95% confidence interval) was significantly higher in patients on systemic versus non-systemic therapy (0.4 [0.2–0.8] versus 0.1 [0.0–0.4]; *p* = 0.0424). Monthly out-of-pocket expenses (USD) were higher in Latvia (mean [SD]: 103.7 [2.64]) versus Estonia (55.6 [1.82]) and Lithuania (53.8 [1.90]). *Conclusions*: Adult AD patients from the Baltic region still face a considerable disease and economic burden, regardless of treatment received. Improved disease management and better access to guideline-recommended advanced systemic therapies are necessary.

## 1. Introduction

Atopic dermatitis (AD) ranks as the 15th leading nonfatal disease globally and is the most prevalent dermatological condition, affecting nearly 20,405 million individuals worldwide [1,2]. The prevalence of AD is estimated at approximately 4% in children and 2% in adults, with substantial variation across countries and ethnic groups [2,3]. Historically, a previous study conducted in Lithuania showed an increasing trend in children aged 6 to 7 years, rising from 1.4% in 1994 to 3.5% in 2002 [4]. Currently, in the Baltic region, the reported prevalence of AD remains relatively low compared to Western and Northern European regions, with 38,434 cases per 100,000 persons in Estonia, 26,664 in Latvia, and 14,777 in Lithuania in 2022 [5,6]. Among adolescents aged 13–14 years in this region, prevalence rates range from 1.8% in Lithuania to 3.4% in Latvia and 8.7% in Estonia [6]. Notably, in Estonia, a markedly higher prevalence of 17.6% has been recorded in adults, whereas no corresponding data are available for Latvia or Lithuania [6].

The biggest organ in the body, the skin, plays a key role in the organism’s response to direct external stimuli—solar radiation, microorganisms, and chemical factors—through its neuro-immuno-endocrine components, thus impacting systemic homeostasis [7]. This provides novel perspectives into the complex pathophysiology of skin diseases, including AD [8,9]. Traditionally seen as a chronic condition of the skin, AD is redefined as a multisystemic disease in which neuro-immune-targeted drugs such as biologics and small molecules reshape treatment prospects [8,10].

AD symptoms vary among individuals and often follow an unpredictable, relapsing-remitting course [11]. Its chronic nature, as well as persistent pruritus, visible skin lesions, sleep disturbances, and the need for treatment, pose a considerable physical, psychological, and economic burden, impacting patients’ quality of life (QoL) [12,13]. The primary goals of AD management are to maintain a disease state in which symptoms are either absent or mild, without interfering with daily activities, and to minimize the need for continuous pharmacological therapy. When remission is not feasible, maintenance of a mild disease state and prevention of acute exacerbations are desired [14].

According to current European guidelines, the management of AD follows a stepwise, individualized approach based on disease severity and patient response. Basic therapy for mild-to-moderate disease relies on the regular use of emollients and moisturizers, topical anti-inflammatories, antimicrobials, antipruritic agents, ultraviolet (UV) phototherapy, and avoidance of provoking factors [15]. Severe cases require systemic therapy, including conventional immunosuppressants, biologics, and Janus kinase (JAK) inhibitors. Systemic corticosteroids are reserved only for short-term use as rescue therapy for acute flares [16,17,18]. Cyclosporine remains the only approved conventional agent for short- to long-term treatment in patients aged ≥16 years, while methotrexate and azathioprine may be considered off-label in selected adult cases for long-term immunosuppression. Among biologics, the monoclonal antibody dupilumab is recommended for patients aged 6 months onward, and lebrikizumab and tralokinumab for those aged 12 years onward. Additionally, three JAK inhibitors—baricitinib, upadacitinib, and abrocitinib—are now approved for moderate-to-severe AD in adults, with expanding indications for pediatric populations: baricitinib for children aged 2 years onward, and upadacitinib and abrocitinib for those aged 12 years and onward [16,17,18].

The introduction of new classes of biologics and novel agents for AD treatment in routine clinical practice has led to substantial variations in treatment strategies and patient outcomes across geographical regions and jurisdictions [19]. Although the European guidelines are generally followed in the Baltic region, recommendations in real-world settings may vary based on national guidelines, accessibility, and reimbursement policies [20,21,22]. Moreover, nationwide registries for AD to monitor treatment patterns, patient outcomes, QOL, and healthcare costs are lacking, which limits the understanding of AD management and outcomes in real-life settings.

To address this gap, the ESSENTIAL AD study characterized the current treatment patterns and clinical status of AD patients treated with systemic, non-systemic, or combination therapies in routine clinical practice in Eastern Europe, the Middle East, and Africa. The overall study findings showed a substantial clinical, QoL, and economic burden among patients, irrespective of treatment type [23]. This manuscript presents findings from the Baltic region.

## 2. Materials and Methods

### 2.1. Study Design and Population

ESSENTIAL AD was a cross-sectional, retrospective chart review, observational study conducted in 15 countries across Eastern Europe, the Middle East, and Africa from 21 September 2021 to 29 June 2022. In the Baltic region, data collection was performed during a single visit to hospitals or office-based specialists at clinical centers in Estonia (n = 2), Latvia (n = 1), and Lithuania (n = 2).

Adult patients (age ≥ 18 years) with a confirmed AD diagnosis receiving any type of treatment in routine settings were eligible for inclusion in the study. Any treatment for AD was allowed by the study protocol: systemic and/or non-systemic therapy, either as monotherapy or in combination. Newly prescribed treatments on the day of the study visit were permitted, as long as the prescription was made before any study-related procedure. All patients provided written informed consent for study participation. Patients receiving any investigational drug, device, or intervention for AD at the time of eligibility assessment, or those who had received an investigational product within one month or five half-lives (whichever was longer) before enrollment, were excluded from the study. Patients were enrolled consecutively at the time of their routine clinic visit.

The following data were collected: demographic and lifestyle characteristics, comorbidities, disease characteristics and severity, and current treatment for AD. Patients receiving any systemic treatment or who initiated it during the study visit were included in the group ‘systemic therapy users’. The others were assigned to the group ‘non-systemic therapy users’. The study investigator at each site entered all data into a password-protected, web-based electronic data capture system. All data were anonymized. More study design details have been published in the primary manuscript [23].

### 2.2. Study Objectives

Primary objectives: to describe the severity of AD symptoms and QoL of patients receiving systemic and/or non-systemic therapy for AD (as monotherapy or in combination) in routine clinical practice.

Secondary objectives: to describe the multi-dimensional profile of AD patients in terms of clinical characteristics, QoL, healthcare resource utilization, work productivity loss, and patient-reported pruritus (itch). Additionally, current AD treatment patterns, disease activity profiles (clinical course and flares), and out-of-pocket expenses were assessed.

### 2.3. Study Outcomes

Primary variables assessed included the Eczema Area and Severity Index (EASI) score (summation of the four regional scores, ranging from 0 to 72) [24], SCORing Atopic Dermatitis (SCORAD) (range between 0 and 100) [25], and the Dermatology Life Quality Index (DLQI) total score (range between 0 and 30, higher scores indicating greater impairment in healthcare-related QoL) [26] at enrollment, including the proportion of patients with different severity categories.

Secondary variables assessed included the Worst Pruritus Numerical Rating Scale (WP-NRS) score (range between 0 = no itch and 10 = worst itch imaginable) [27]; the Work Productivity and Activity Impairment—Atopic Dermatitis (WPAI-AD) total score (higher numbers indicating greater impairment and less productivity over the past 7 days) [28,29]; current treatments, including clinical courses of AD (seasonal, episodic [moderate or severe], consistent, and consistent with flares); occurrences of flares in the past 12 months; routine healthcare visits; acute care visits due to AD in the last 12 months; and out-of-pocket expenses.

### 2.4. Statistical Analysis

Descriptive statistics were applied to the full analysis set (FAS), which included all patients meeting the study inclusion criteria and having no exclusion criteria. Demographic, clinical characteristics, treatment patterns, and QoL variables were summarized. Quantitative variables were reported as the number of patients, mean (standard deviation [SD]), median (interquartile range [IQR]), and 95% confidence intervals (CI) for mean or median, as appropriate. Categorical variables were reported as the number (proportion) of patients within each category. Corresponding scores recorded at enrollment and initiation of the current treatment (when available) were reported as the number and percentage of patients with 95% CI.

Subgroup analyses were conducted based on treatment type (systemic, including combination treatment, versus non-systemic). Comparisons between subgroups were performed using the Kruskal–Wallis test for continuous variables and a Pearson’s chi-square test for categorical variables. Negative binomial regression models were used for the estimation of rates and associated measures.

The significance level was set to *p* < 0.05.

## 3. Results

### 3.1. Patient Characteristics and Treatment Patterns

In the Baltic region, 50 AD patients were enrolled in the ESSENTIAL AD study (20 [40.0%] in Estonia, 10 [20.0%] in Latvia, and 20 [40.0%] in Lithuania), and all were included in the FAS. Proportion of patients was evenly distributed between Systemic therapy and Non-systemic therapy groups (Table 1).

Mean (SD) age at study enrollment was 33.6 (11.67) years. AD patients on systemic therapy at study enrollment were significantly younger at AD diagnosis than those receiving non-systemic therapy, with a mean age of 5.96 versus 14.31 years, respectively. More than half (30 [60%]) of patients were female, and all but one were Caucasian (49 [98.0%]). The most commonly reported comorbidities were allergic rhinitis and asthma. Most frequently used systemic therapy during the 12 months before the study visit was cyclosporine (Table 1). Symptomatic treatment with antihistamines was reported in 17 [34.0%] patients: 8 (32%) in the Systemic therapy group and 9 (36.0%) in the Non-systemic therapy group.

**Figure 1:**
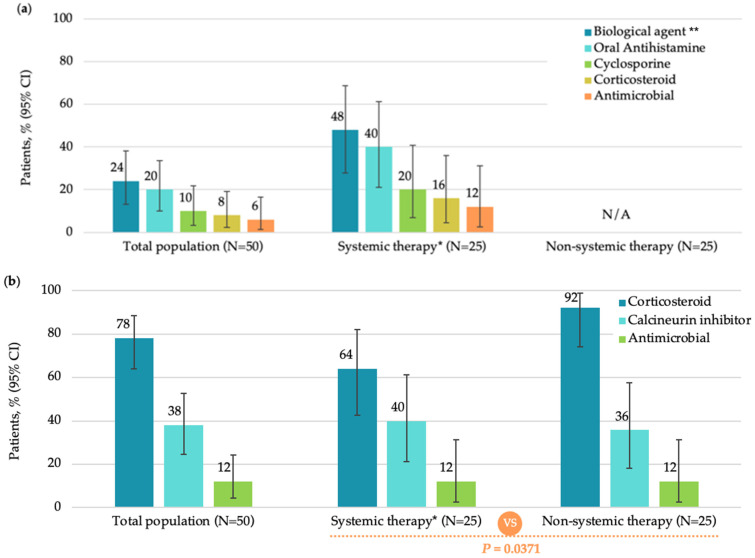

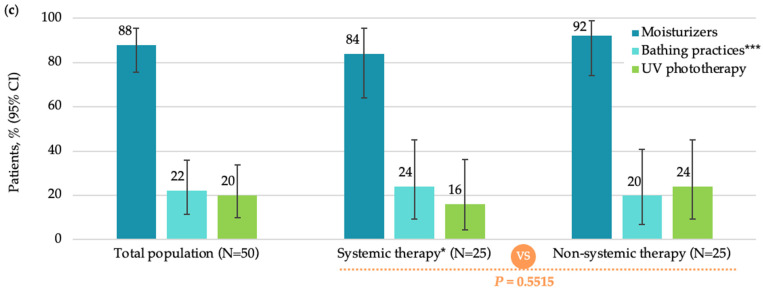
Current treatments in the total population, systemic therapy subgroup, and non-systemic therapy subgroup (FAS): (**a**) systemic monotherapy or in combination; (**b**) topical monotherapy or in combination; (**c**) non-pharmacological therapy. CI, confidence interval; FAS, full analysis set; N, total number of patients; UV, ultraviolet. Note: Patients in the Systemic therapy group may have received oral antihistamines as adjuvant therapy per medical needs. * Including combination therapy; ** Dupilumab administered in all 12 patients on biologic therapy; *** Including additives.

The median (IQR) time since the prescription of current treatment was significantly longer in patients receiving systemic therapy compared to those receiving non-systemic therapy (4.50 [0.39, 14.82] months versus 0.11 [0.00–2.23] months; *p* = 0.0009). In the overall population (FAS), the most commonly used current pharmacological systemic agents were biologics (12 [24.0%]). No patient received JAK inhibitors at study enrollment (Figure 1a).

Most patients in FAS (46 [92.0%]) were treated with pharmacological non-systemic (topical) treatment, among which, corticosteroids (39 [78.0%]) and calcineurin inhibitors (19 [38.0%]) were most frequently used (Figure 1b). The most commonly used non-pharmacological therapy was moisturizers (44 [88.0%]) (Figure 1c).

### 3.2. Primary Outcomes: EASI, SCORAD, and DLQI Scores at Study Visit

**Figure 2:**
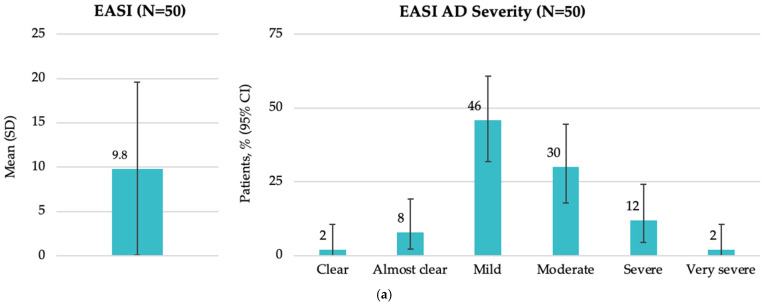

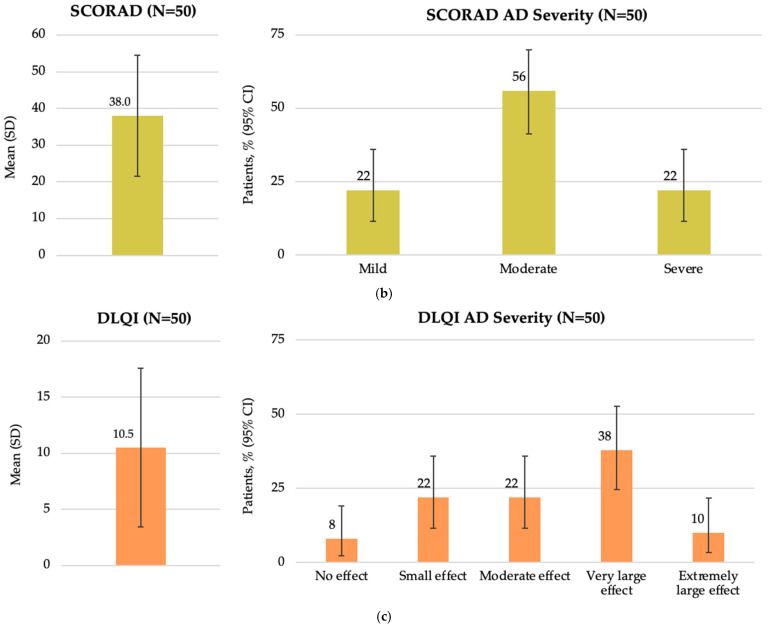
Disease characteristics by EASI, SCORAD, and DLQI total scores at enrollment in the Baltic population (FAS): (**a**) overall EASI score and patient distribution by EASI AD severity; (**b**) overall SCORAD score and patient distribution by SCORAD AD severity; and (**c**) overall DLQI score and patient distribution by DLQI AD severity. AD, atopic dermatitis; CI, confidence interval; DLQI, Dermatology Life Quality Index; EASI, Eczema Area and Severity Index; FAS, full analysis set; N, total number of patients; SCORAD, SCORing Atopic Dermatitis; SD, standard deviation.

The mean (SD) EASI score at study visit was 9.8 (9.76), with a median of 6.1 (IQR: 3.6, 15.0). According to EASI severity categories, most patients had mild (46.0%) or moderate (30.0%) disease (Figure 2a). The mean (SD) SCORAD score was 38.0 (16.5), with a median of 35.8 (IQR: 30.6, 47.1). Most patients (56.0%) had moderate disease as assessed by SCORAD (Figure 2b). The mean (SD) DLQI score was 10.5 (7.1), with a median of 9.5 (IQR: 5.0, 14.0). The impact of AD on patient QoL was assessed by the DLQI, showing that more than one-third of patients (38.0%) reported a very large impact (Figure 2c).

**Figure 3:**
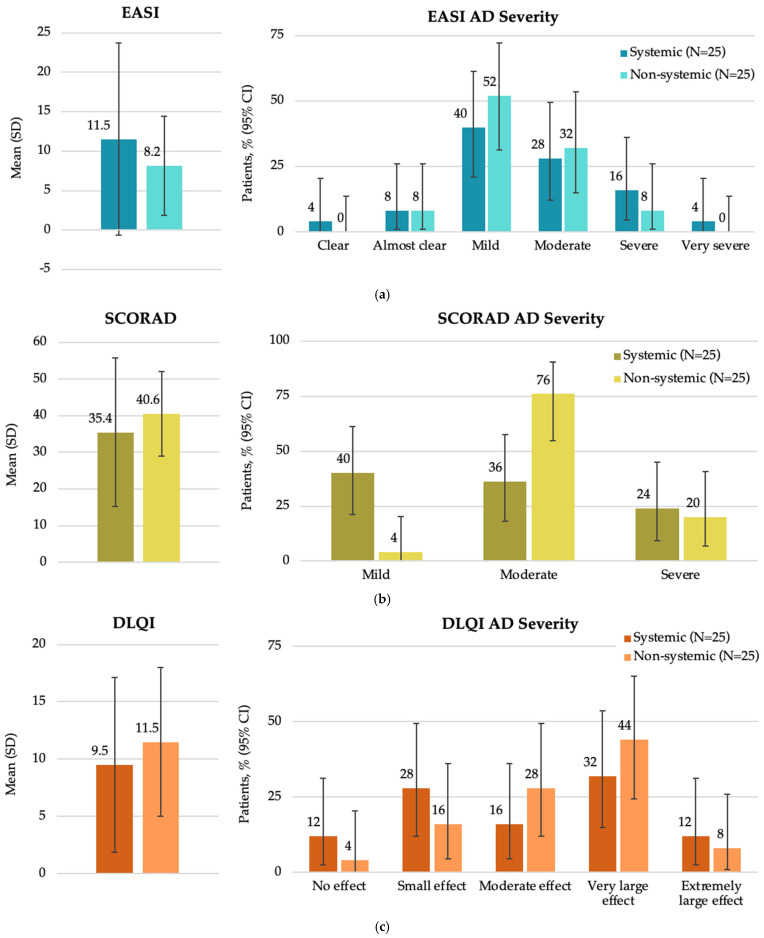
Disease characteristics by EASI, SCORAD, and DLQI total scores at enrollment in the Baltic population by systemic and non-systemic therapy subgroups (FAS): (**a**) overall EASI score and patient distribution by EASI AD severity; (**b**) overall SCORAD score and patient distribution by SCORAD AD severity; (**c**) overall DLQI score and patient distribution by DLQI AD severity. AD, atopic dermatitis; CI, confidence interval; DLQI, Dermatology Life Quality Index; EASI, Eczema Area and Severity Index; FAS, full analysis set; N, total number of patients; SCORAD, SCORing Atopic Dermatitis; SD, standard deviation.

At study visit, there were no significant differences among study groups in terms of the EASI score (*p* = 0.7636), SCORAD (*p* = 0.2563) and DLQI (*p* = 0.1962) (Figure 3). Additionally, no significant differences were observed in patient distributions based on EASI and DLQI severity categories (*p* = 0.6808 and *p* = 0.507, respectively). A higher proportion of patients receiving systemic therapy had mild disease, as assessed by SCORAD, while a higher proportion of patients receiving non-systemic therapy had moderate disease (*p* = 0.0040).

### 3.3. Secondary Outcomes: WP-NRS, WPAI-AD, Clinical Disease Characteristics, and Economic Burden of AD at Study Visit

At study visit, the overall mean (SD) WP-NRS score was 5.7 (2.48), with a median of 6.0 (4.0, 8.0), suggesting the presence of moderate-to-severe itch in patients with AD. Among the 41 employed patients, the mean (SD) work productivity loss was 32.3% (29.85%). Interference with daily activity averaged 32.6% (SD 31.61%) across the total study population. Regarding the most common disease courses, 34% of patients reported clinical characteristics consistent with flares, and 24% reported episodic moderate disease. During the 12 months before the study visit, most patients (90%) reported the occurrence of flares, with a median (IQR) of 2.5 (1.0, 6.0) flares and a median (IQR) duration of 14.0 (7.0, 30.0) days (Table 2). Disease burden was generally comparable between groups, except for itch severity (as assessed by WP-NRS score). Patients treated with non-systemic therapy reported significantly higher WP-NRS scores compared to those receiving systemic therapy (*p* = 0.0047) (Table 2).

Healthcare resource utilization over the preceding year included a median (IQR) of 5.0 (2.0, 8.0) routine healthcare visits (Table 3). More acute/emergency visits were reported in patients receiving systemic therapy; however, data was available for a lower number of patients, and it did not reach the significance threshold (Table 3). Hospitalization rate was significantly higher in patients on systemic therapy versus those on non-systemic therapy (0.4 [95% CI: 0.2–0.8] versus 0.1 [95% CI: 0.0–0.4]; *p* = 0.0424).

Monthly out-of-pocket expenses and extra amount spent as a result of AD were higher in Latvia (mean [SD] 103.7 [2.64] USD) compared to Estonia (mean [SD] 55.6 [1.82] USD) and Lithuania (53.8 [1.90] USD) in the total population. The economic burden was generally similar between patients treated with systemic therapy and those treated with non-systemic therapy in all three countries. Although monthly expenses tended to be higher for patients receiving systemic therapy compared to those receiving non-systemic therapy in Latvia, the difference did not reach statistical significance (Table 4).

## 4. Discussion

The current analysis of the ESSENTIAL AD study data provides the first real-world evidence on the disease and economic burden, as well as treatment patterns in adult patients managed with systemic and/or non-systemic therapy in routine clinical settings in the Baltic region (Estonia, Latvia, and Lithuania).

The study population was generally in line with the AD patient profile. Patients were relatively young at enrollment (33.6 years), consistent with the overall ESSENTIAL AD cohort (36.3 years) [23], the Portugal and Greece cohorts from the MEASURE-AD study (36.3 years) [30] and the Spanish cohort from the APOLO study (33.1 years) [31], but slightly younger than in other real-world studies (37.2 to 50.7 years) [32,33,34,35,36]. A predominance of female patients (60%) was observed, in line with the overall ESSENTIAL AD cohort (57%) [23]. In contrast, other real-world studies have reported a higher proportion of males (51% to 61%) [30,31,32,35,36,37]. While the clinical relevance of this observation is unclear, it may reflect higher healthcare-seeking behavior among women in the Baltic region and other settings of the ESSENTIAL AD study [38]. The most frequently reported atopic comorbidities were allergic rhinitis (24.0%) and asthma (20%), similar to most real-world studies [23,30,31,34,35,37,39] and consistent with well-established disorders that are associated with AD and are considered components of the atopic march [40].

At study enrollment, the proportion of patients on modern therapies, such as biologic agents, increased to 48% (12/25) from 12% (3/25) one year earlier, indicating a recent rise in biological therapy prescribing. Although their overall use remained relatively limited, it was higher than in the overall ESSENTIAL AD cohort (22.3%) [23] and comparable to that in other recent real-world studies (37.7–56.3%) [30,32,35]. No patients were receiving JAK inhibitors as reimbursement was still pending at the time of the study, and 16% (4/25) were still receiving systemic corticosteroids, a pattern consistent with findings from the overall cohort and other real-world studies [23,30,32,35,37], despite recommendations against routine use of systemic corticosteroids in EuroGuiDerm and local guidelines [16,17,18,20,21]. These results underscore the need to improve access to modern therapies in the Baltic countries, and also in other European settings. Additionally, only 84% of patients reported regular use of emollients, similar to the overall cohort [23] and another real-world study [31].

Despite ongoing therapies, 78% and 42% of patients had moderate-to-severe disease based on SCORAD and EASI scores, respectively. The reported flare rate averaged 3.6 in the preceding year, with a mean duration of flares of 46.0 days. The mean [SD] DLQI was 10.5 [7.1], with nearly half (48%) of patients reporting a very large or extremely large negative impact on QoL (DLQI ≥ 11), falling within the range reported in other real-world studies (27.4–65.5%) [31,35,36]. These findings suggest that many patients continue to experience significant disease activity and impaired QoL, despite ongoing therapy, potentially indicating suboptimal treatments or inherently difficult-to-control cases. In contrast, a study assessing the impact of AD on QoL in the Lithuanian pediatric population found a moderate effect of AD on dermatology-related QoL (mean [SD] 6.3 [5.6]), a difference that could be attributed to the different population age [41]. The substantial disease burden observed in our cohort is consistent with the one observed in the larger ESSENTIAL AD study conducted in 15 countries from Eastern Europe, the Middle East, and Africa [23], and in the global MEASURE-AD study including adult and adolescent patients in 28 countries across Europe, American continent, Australia and New Zealand, Asia and Middle East [30,32,35]. Similar unmet clinical needs and impaired QoL have also been reported in other real-world studies conducted in Europe, the United States, Canada, and Asia Pacific region [33,34,36,37,39].

A tendency for a higher EASI score was observed in patients receiving systemic versus non-systemic therapy (11.5 versus 8.2, respectively), while a higher SCORAD score was noted in those receiving non-systemic versus systemic treatment (40.6 versus 35.4, respectively) at enrollment. The discrepancy in SCORAD outcomes observed between the Baltic cohort and the overall study cohort [23] and another real-world study [33] may be attributed to the SCORAD evaluation system, which captures additional clinical features such as skin dryness and crusting. Patients receiving systemic therapy may adhere more consistently to adjunctive topical care, including emollients, which could influence these parameters and partially account for the observed difference. The fact that a higher proportion of patients (40%) receiving systemic therapy had mild disease as assessed by SCORAD, while a higher proportion of patients receiving non-systemic therapy had moderate disease (76%), is likely reflecting the use of systemic treatment before study enrolment and response rather than preferential use of systemic agents in milder AD cases. Patients receiving systemic therapy also had a longer duration of current treatment compared to those receiving non-systemic therapy (4.50 vs. 0.11 months, respectively). Notably, patients with severe AD undergoing systemic treatment had higher AD severity scores compared to those receiving non-systemic treatments (EASI: 16 versus 8; SCORAD: 24 versus 20, respectively), indicating that more severe cases are being treated with systemic therapies. This finding underscores the need for improved AD management strategies and access to effective treatments for AD in the region.

In addition to the clinical burden and influence on QoL as shown by the number of flares in the past year and DLQI, WP-NRS and WPAI-AD scores, a substantial economic impact was observed in the Baltic countries. Out-of-pocket monthly healthcare-related expenses due to AD were highest in Latvia (mean [SD]: 103.7 [2.64] USD), followed by Estonia (55.6 [1.82] USD) and Lithuania (53.8 [1.90] USD). This pattern was consistent across countries, with no statistically significant difference between patients receiving systemic versus non-systemic therapies. However, in Latvia, expenses tended to be higher in the systemic therapy group, likely reflecting differences in national reimbursement policies.

Across the Baltic region, clinical practice patterns and access to newly approved advanced AD therapies are governed by country-specific reimbursement policies. Estonia follows European guidelines for AD management, with reimbursement for targeted therapies available when the EASI score is ≥16, the DLQI score is ≥10, and an inadequate response is observed after 8–12 weeks of systemic cyclosporine or when cyclosporine is contraindicated or not tolerated. Dermatovenerologists, allergists, or pediatricians can prescribe these agents. In Latvia, national guidelines have been established [21], with prescribing criteria largely aligned with those in Estonia. However, reimbursement policies differ in that topical treatments (i.e., mometasone furoate, pimecrolimus, and tacrolimus), UV phototherapy, and systemic upadacitinib are reimbursed for both adults and children, while dupilumab is reimbursed exclusively for pediatric patients. Other systemic agents (i.e., corticosteroids, methotrexate, and cyclosporine) are not reimbursed, contributing to the higher out-of-pocket expenses reported in this study, particularly among patients requiring systemic therapies. In Lithuania, national guidelines based on European recommendations were first introduced in 2019 [20] and updated in 2024 [22]. All targeted therapies (i.e., dupilumab, upadacitinib, and baricitinib) are reimbursed for adults with severe AD for whom cyclosporine therapy is inappropriate, contraindicated, or does not achieve the goal of treatment (the latter criterion applies to upadacitinib only), and upadacitinib additionally for adolescents aged ≥12 years. Dermatovenerologists or allergist-clinical immunologists can prescribe all these agents, and pediatric allergists are additionally authorized to prescribe upadacitinib. Treatment discontinuation is recommended after 16 weeks (dupilumab, baricitinib) and 12 weeks (upadacitinib) if no benefit is observed. Topical treatments (i.e., pimecrolimus, tacrolimus) are reimbursed for children with moderate-severe AD when glucocorticoids are contraindicated due to adverse effects or have failed to achieve treatment goals after 3 months of use. Additional systemic and topical agents (i.e., fluticasone, methylprednisolone, mometasone furoate, betamethasone, and cyclosporine) are reimbursed for severe AD in both adults and children, while fusidic acid plus hydrocortisone is only reimbursed for pediatric patients.

These discrepancies in clinical practice and access to reimbursed treatments across the Baltic countries likely contribute to the observed differences in patient-reported economic burden and may further impact clinical outcomes and QoL. This is a global issue, which is why the GA^2^ LEN ADCARE initiative, a concept for integrated care pathways for atopic dermatitis, is ongoing and making continuous efforts to bridge the gap between existing AD treatment evidence-based guidelines and expert opinion based on daily practice to provide pragmatic and practical support for optimally managing the disease and its comorbidities worldwide [19].

The strength of the current study is that it provides recent, reliable, real-world evidence on the burden of AD and treatment patterns in the Baltic countries. However, it has limitations typical of real-world studies with a cross-sectional, single-visit design, such as patient selection, recall bias, and misclassification. Due to its descriptive design and analysis, true differences between treatment groups could not be adequately identified in this dataset. To mitigate selection bias, consecutive patients were enrolled at each study site. While this is the first study in the AD adult patient population in the Baltics, the small sample sizes in each country and across groups, and the homogeneity of the population (all patients, except one, were Caucasian) do not allow for generalizability. At the time of the study, access to biologics and JAK inhibitors was limited, and reimbursement policies varied across countries, potentially introducing selection bias linked to treatment availability and local clinical practices.

## 5. Conclusions

This analysis of data from the ESSENTIAL AD study showed that adult patients with AD from the Baltic countries continue to experience a considerable disease burden, regardless of whether they receive systemic or non-systemic therapies. While the uptake of biologic agents is increasing, with 1 in 2 patients receiving systemic therapy with biologicals, 1 in 6 patients still receive systemic corticosteroids. These findings highlight unmet needs for optimal disease control and improved access to guideline-recommended advanced systemic therapies for the management of AD. The high economic burden observed in these countries may further affect patient outcomes and QoL, underscoring the need for cost-effective treatments and healthcare strategies that can alleviate this burden.

These findings highlight the importance of implementing harmonized, evidence-based treatment pathways and reimbursement policies to optimize care and reduce the overall disease and economic burden in the Baltic region.

## Figures and Tables

**Table 1:** Baseline patient demographics and clinical characteristics (FAS).

	Total Population (N = 50)	Systemic Therapy * Users (N = 25)	Non-Systemic Therapy Users (N = 25)	Comparison of Subgroups *p* Value
Country, n (%)				
Estonia	20 (40.0)	10 (40.0)	10 (40.0)	>0.9999
Latvia	10 (20.0)	5 (20.0)	5 (20.0)	
Lithuania	20 (40.0)	10 (40.0)	10 (40.0)	
Age at consent, years				
Mean (SD)	33.6 (11.67)	33.0 (11.66)	34.3 (11.87)	0.7781
Median (IQR)	32.5 (24.0, 40.0)	27.0 (24.0, 40.0)	37.0 (23.0, 40.0)	
Age at diagnosis, years				**0.0436**
Mean (SD)	10 (13.395)	5.96 (7.818)	14.31 (16.284)	
Median (IQR)	3.56 (0.53, 18.02)	1.05 (0.00, 12.55)	7.66 (2.51, 22.43)	
Female, n (%)	30 (60.0)	10 (40.0)	20 (80.0)	**0.0039**
Race, n (%)				
Caucasian	49 (98.0)	24 (96.0)	25 (100)	0.3124
Asian	1 (2.0)	1 (4.0)	0 (0.0)	
Employed, n (%)	41 (82.0)	20 (80.0)	21 (84.0)	0.3679
Health insurance coverage, n (%)				
Public	49 (98.0)	25 (100)	24 (96.0)	0.3124
Public–private mix	1 (2.0)	0 (0.0)	1 (4.0)	
Time since AD diagnosis,				
Years				
Patients with data available	n = 39	n = 19	n = 20	
Mean (SD)	21.79 (14.842)	25.08 (14.589)	18.67 (14.760)	0.1292
Median (IQR)	21.40 (9.60, 33.08)	24.46 (12.05, 35.55)	14.71 (6.06, 32.30)	
Previous therapy (last 12 months before study visit), n (%)				
Systemic therapy	28 (56.0)	18 (72.0)	10 (40.0)	0.0227
Cyclosporine	10 (20.0)	9 (36.0)	1 (4.0)	
Corticosteroid	7 (14.0)	7 (28.0)	0 (0.0)	
Biologic agent ^	3 (6.0)	3 (12.0)	0 (0.0)	
JAK inhibitor ^^	1 (2.0)	1 (4.0)	0 (0.0)	
Antimicrobial	1 (2.0)	1 (4.0)	0 (0.0)	
Topical therapy	40 (80.0)	18 (72.0)	22 (88.0)	0.1573
Corticosteroid	36 (72.0)	16 (64.0)	20 (80.0)	
Calcineurin inhibitor	24 (48.0)	14 (56.0)	10 (40.0)	
Antimicrobial	5 (10.0)	2 (8.0)	3 (12.0)	
Non-pharmacological treatment	43 (86.0)	20 (80.0)	23 (92.0)	0.2214
Moisturizers	42 (84.0)	20 (80.0)	22 (88.0)	
Phototherapy	12 (24.0)	7 (28.0)	5 (20.0)	
Bathing Practices	11 (22.0)	7 (28.0)	4 (16.0)	
Wet wrap therapy	3 (6.0)	1 (4.0)	2 (8.0)	
Other	1 (2.0)	1 (4.0)	0 (0.0)	
Any comorbidity	25 (50.0)	10 (40.0)	15 (60.0)	0.1573
Most common comorbidities **				
Respiratory, thoracic and mediastinal disorders	17 (34.0)	7 (28.0)	10 (40.0)	0.3705
Allergic rhinitis	12 (24.0)	4 (16.0)	8 (32.0)	
Asthma	10 (20.0)	5 (20.0)	5 (20.0)	
Pulmonary sarcoidosis	1 (2.0)	0 (0.0)	1 (4.0)	
Metabolism and nutrition disorders	3 (6.0)	3 (12.0)	0 (0.0)	0.2347
Dyslipidemia	1 (2.0)	1 (4.0)	0 (0.0)	
Hypercholesterolemia	1 (2.0)	1 (4.0)	0 (0.0)	
Obesity	1 (2.0)	1 (4.0)	0 (0.0)	
Type 2 diabetes mellitus	1 (2.0)	1 (4.0)	0 (0.0)	

AD, atopic dermatitis; N, total number of patients; n (%), number (percentage) of patients within a specific category; IQR, interquartile range; SD, standard deviation; UV, ultraviolet. Note: Bolded value indicates statistical significance (*p* < 0.05). * Including combination therapy; ** Occurring in ≥6% of patients in either subgroup; ^ Dupilumab administered in all 3 patients on biologic therapy; ^^ Abrocitinib.

**Table 2:** AD burden in the overall Baltic population and by systemic and non-systemic users at study visit (FAS). Number of patients with data available for analysis is reported for specific items, where not all patients had data collected.

	Total Population (N = 50)	Systemic Therapy Users (N = 25)	Non-Systemic Therapy Users (N = 25)	*p* Value
WP-NRS				
Mean (SD)	5.7 (2.48)	4.7 (2.44)	6.6 (2.14)	**0.0047**
Median (IQR)	6.0 (4.0, 8.0)	4.0 (3.0, 7.0)	7.0 (6.0, 8.0)	
WPAI-AD				
Absenteeism, %				
Patients with data available	n = 41	n = 21	n = 20	
Mean (SD)	6.9 (14.88)	8.5 (19.01)	5.2 (8.96)	0.6497
Median (IQR)	0.0 (0.0, 7.0)	0.0 (0.0, 0.0)	0.0 (0.0, 9.4)	
Presenteeism, %				
Patients with data available	n = 43	n = 21	n = 22	
Mean (SD)	31.6 (30.07)	28.1 (28.04)	35.0 (32.18)	0.5142
Median (IQR)	30.0 (0.0, 50.0)	20.0 (10.0, 50.0)	30.0 (0.0, 60.0)	
Overall work productivity impairment *, %				
Patients with data available	n = 41	n = 21	n = 20	0.8536
Mean (SD)	32.3 (29.85)	33.2 (30.74)	31.4 (29.65)	
Median (IQR)	30.0 (0.0, 50.0)	20.0 (10.0, 50.0)	30.0 (0.0, 49.2)	
Daily activity impairment, %	n = 50	n = 25	n = 25	
Mean (SD)	32.6 (31.61)	29.2 (27.07)	36.0 (35.82)	0.7832
Median (IQR)	20.0 (0.0, 60.0)	20.0 (10.0, 50.0)	30.0 (0.0, 80.0)	
Clinical course of AD, n (%)				
Seasonal	7 (14.0)	2 (8.0)	5 (20.0)	0.3452
Episodic (moderate)	12 (24.0)	5 (20.0)	7 (28.0)	
Episodic (severe)	9 (18.0)	6 (24.0)	3 (12.0)	
Consistent	5 (10.0)	4 (16.0)	1 (4.0)	
Consistent with flares	17 (34.0)	8 (32.0)	9 (36.0)	
Flares, past 12 months, n (%)	45 (90.0)	22 (88.0)	23 (92.0)	0.6374
Number of flares, past 12 months				
Mean (SD)	3.6 (3.52)	3.0 (2.30)	4.2 (4.38)	0.2864
Median (IQR)	2.5 (1.0, 6.0)	3.0 (1.0, 5.0)	2.0 (1.0, 6.0)	
Estimated flare rate in the past 12 months, mean (95% CI) **	3.6 (2.8–4.6)	3.0 (2.1–4.3)	4.2 (3.0–6.0)	0.1636
Average duration of flares, days				
Patients with data available	n = 45	n = 22	n = 23	
Mean (SD)	46.0 (86.50)	35.6 (75.86)	56.0 (96.22)	0.6568
Median (IQR)	14.0 (7.0, 30.0)	14.0 (7.0, 30.0)	14.0 (7.0, 60.0)	

AD, atopic dermatitis; CI, confidence interval; FAS, full analysis set; IQR, interquartile range; N, total number of patients; n, number of patients within a specific category; SD, standard deviation; WPAI, Work Productivity and Activity Impairment; WP-NRS, worst pruritus–numeric rating scale. Note: Bolded value indicates statistical significance (*p* < 0.05). * Percent overall work impairment (work productivity loss, or absenteeism + presenteeism) at study visit. ** Estimated using negative binomial distribution.

**Table 3:** Healthcare resource utilization in the overall Baltic AD population and by systemic and non-systemic users at study visit (FAS). Number of patients with data available for analysis is reported for specific items, where not all patients had data collected.

	Total Population (N = 50)	Systemic Therapy Users (N = 25)	Non-Systemic Therapy Users (N = 25)	*p* Value
Number of routine healthcare visits, past 12 months				
Patients with data available	n = 45	n = 22	n = 23	0.1412
Mean (SD)	6.8 (6.91)	6.7 (4.10)	6.8 (8.91)	
Median (IQR)	5.0 (2.0, 8.0)	6.0 (4.0, 9.0)	4.0 (2.0, 8.0)	
Estimated routine healthcare visit rate, past 12 months, mean (95% CI) *	6.8 (5.2–8.9)	6.7 (4.6–9.9)	6.8 (4.7–9.9)	0.9574
Number of acute/emergency healthcare visits, past 12 months				
Patients with data available	n = 34	n = 16	n = 18	
Mean (SD)	0.9 (2.11)	1.4 (2.87)	0.4 (0.92)	0.1895
Median (IQR)	0.0 (0.0, 1.0)	0.0 (0.0, 1.0)	0.0 (0.0, 0.0)	
Estimated acute/emergency healthcare visit rate in the past 12 months, mean (95% CI) *	0.9 (0.4–2.1)	1.4 (0.5–4.5)	0.4 (0.1–1.4)	0.1480

AD, atopic dermatitis; CI, confidence interval; FAS, full analysis set; IQR, interquartile range; N, total number of patients; n, number of patients within a specific category; SD, standard deviation. Note: Statistical significance threshold was set at *p* < 0.05. * Estimated using negative binomial distribution.

**Table 4:** Out-of-pocket expenses due to AD by country (FAS).

Expenses in USD		Total Population	Systemic Therapy Users	Non-Systemic Therapy Users	*p* Value
Monthly healthcare-related expenses					
Estonia					
	Number of patients	n = 20	n = 10	n = 10	0.7327
	Mean (SD)	55.6 (1.82)	56.5 (1.35)	54.7 (2.26)	
	Median (IQR)	56.7 (44.8, 73.1)	56.2 (52.5, 56.7)	59.9 (22.5, 113.3)	
Latvia					
	Number of patients	n = 10	n = 5	n = 5	0.3457
	Mean (SD)	103.7 (2.64)	136.8 (2.45)	78.5 (2.88)	
	Median (IQR)	184.2 (33.7, 224.7)	215.3 (153.0, 226.7)	78.6 (33.7, 223.3)	
Lithuania					
	Number of patients	n = 20	n = 10	n = 10	0.7906
	Mean (SD)	53.8 (1.90)	56.2 (1.91)	51.5 (1.96)	
	Median (IQR)	56.2 (34.0, 79.3)	51.0 (34.0, 90.7)	56.2 (28.3, 68.0)	
Monthly extra amount spent on everyday necessities					
Estonia					
	Number of patients	n = 20	n = 10	n = 10	0.9095
	Mean (SD)	43.7 (7.85)	64.0 (1.88)	29.8 (17.63)	
	Median (IQR)	56.0 (38.0, 113.3)	59.4 (34.0, 113.3)	51.7 (42.0, 113.3)	
Latvia					
	Number of patients	n = 10	n = 5	n = 5	
	Mean (SD)	4.3 (70.03)	2.0 (131.13)	9.1 (50.63)	0.9153
	Median (IQR)	23.7 (0.0, 113.3)	24.9 (0.0, 113.3)	22.5 (22.5, 56.2)	
Lithuania					
	Number of patients	n = 20	n = 10	n = 10	
	Mean (SD)	20.8 (7.56)	13.2 (13.73)	32.9 (3.14)	0.9092
	Median (IQR)	28.1 (11.3, 56.7)	28.3 (11.3, 40.2)	28.1 (11.2, 56.7)	

AD, atopic dermatitis; FAS, full analysis set; IQR, interquartile range; N, total number of patients; n, number of patients within a specific category; SD, standard deviation; USD, United States dollar.

## Data Availability

AbbVie is committed to responsible data sharing regarding the clinical trials we sponsor. This includes access to anonymized, individual, and trial-level data (analysis datasets), as well as other information (e.g., protocols, clinical study reports, or analysis plans), as long as the trials are not part of an ongoing or planned regulatory submission. This includes requests for clinical trial data for unlicensed products and indications. These clinical trial data can be requested by any qualified researchers who engage in rigorous, independent, scientific research, and will be provided following review and approval of a research proposal, Statistical Analysis Plan (SAP), and execution of a Data Sharing Agreement (DSA). Data requests can be submitted at any time after approval in the US and Europe and after acceptance of this manuscript for publication. The data will be accessible for 12 months, with possible extensions considered. For more information on the process or to submit a request, visit the following link: https://vivli.org/ourmember/abbvie/. Then select “Home”.

## Abbreviations

The following abbreviations are used in this manuscript:

AD	Atopic dermatitis
CI	Confidence interval
DLQI	Dermatology Life Quality Index
EASI	Eczema Area and Severity Index
FAS	Full analysis set
IQR	Interquartile range
JAK	Janus kinase
N	Total number of patients
n	number of patients within a specific category
QoL	Quality of life
SCORAD	SCORing Atopic Dermatitis
SD	Standard deviation
UV	Ultraviolet
WPAI-AD	Work Productivity and Activity Impairment—Atopic Dermatitis
WP-NRS	Worst Pruritus Numerical Rating Scale
